# Association Between Mineral Intake and Cognitive Performance in Spanish Adults with Overweight and Obesity: A Cross-Sectional Study

**DOI:** 10.3390/nu18071129

**Published:** 2026-03-31

**Authors:** Mario Tomé-Fernández, Laura Martín-Manchado, Miriam Sánchez-Sansegundo, Ana Zaragoza-Martí, Jorge Azorín-López, José Antonio Hurtado-Sánchez

**Affiliations:** 1 Department of Health Psychology, Faculty of Health Science, University of Alicante, 03690 Alicante, Spain; mario.tome@ua.es (M.T.-F.); ja.hurtado@ua.es (J.A.H.-S.); 2 Department of Nursing, Faculty of Health Science, University of Alicante, 03690 Alicante, Spain; laura.martinm@ua.es (L.M.-M.); ana.zaragoza@ua.es (A.Z.-M.); 3 Alicante Health and Biomedical Research Institute, ISABIAL Foundation, 03010 Alicante, Spain; 4 Department of Computer Science and Technology, University of Alicante, 03690 Alicante, Spain; jazorin@ua.es

**Keywords:** cognitive performance, executive function, iron, mineral intake, micronutrients, nutrition, zinc

## Abstract

**Background/Objectives**: While adequate mineral intake is essential for brain health and cognitive function across the lifespan, the potential impact of excessive consumption remains underexplored. This study aimed to examine the association between dietary intake of selected minerals, with particular focus on iron and zinc, and cognitive performance in Spanish adults with obesity, particularly in executive-related domains such as reasoning, cognitive flexibility, and working memory. **Methods**: A cross-sectional study was conducted in 230 Spanish adults (18–65 years) from the Tech4Diet-Person project. Sociodemographic, dietary, and cognitive data were collected between 2021 and 2024. Cognitive function was assessed using the validated computerized CogniFit battery, and mineral intake was estimated through a food frequency questionnaire (93 items). Individuals with neurological, metabolic, or psychiatric disorders, as well as pregnant or lactating women, were excluded. **Results**: Participants had a mean age of 45.91 (±9.92) years. Nominal differences in mineral intake were observed across specific executive cognitive domains. Higher dietary iron intake was associated with lower performance in reasoning and cognitive flexibility, while higher zinc intake was associated with lower working memory performance. In adjusted logistic regression models, higher iron intake was independently associated with increased odds of low reasoning performance (OR = 1.25; *p* = 0.006), and higher zinc intake was associated with increased odds of low working memory performance (OR = 1.36; *p* = 0.024), after controlling for age, educational level, BMI, and total energy intake. **Conclusions**: Higher self-reported intake of iron and zinc showed nominal associations with lower performance in specific executive domains. These findings should be considered exploratory and require confirmation in longitudinal and biomarker-based studies.

## 1. Introduction

Nutrition plays a fundamental role in the development and maintenance of brain function, with profound implications for cognitive well-being across the lifespan [1,2]. An adequate intake of nutrients is essential for optimal brain performance, as it influences neurotransmitter synthesis, neuronal activity, and the structural integrity of cell membranes [2]. Within this context, minerals stand out for their critical role in neurological health. Essential minerals such as iron (Fe), zinc (Zn), magnesium (Mg), copper (Cu), and selenium (Se) serve as cofactors in numerous biochemical processes vital to brain function. For instance, iron is indispensable for normal neurocognitive development and myelin production [3]; zinc is associated with attention, learning, and memory [4]; and magnesium is crucial for nervous system function and processes such as memory and attention, partly because magnesium deficiency has been linked to chronic low-grade brain inflammation [5].

Several studies have documented associations between specific mineral levels and cognitive performance at different stages of the life course. For example, lower magnesium intake has been associated with an increased risk of cognitive decline in older adults, due to its role in oxidative stress and neuroinflammation [5,6]. In this regard, data from a large cohort of 2508 adults in the NHANES study revealed that individuals with high daily magnesium intake exhibited better global cognitive test scores, particularly among women and individuals with adequate vitamin D levels [7]. Similarly, among older adults, adequate copper intake (around 1.3 mg/day) has been associated with reduced cognitive decline over time, suggesting a potential neuroprotective role for this mineral [8]. These findings highlight the brain’s sensitivity to variations in mineral availability and support the notion that even subtle imbalances can have significant consequences for cognitive function.

Historically, much of the research in nutritional science has focused on the detrimental effects of mineral deficiencies, consistently documenting their negative impact on brain development, neuronal plasticity, and cognitive performance [9,10]. This perspective has informed the design of nutritional interventions and public health policies aimed at preventing deficiency states, especially during critical periods such as childhood and old age. However, the consequences of nutritional excess, particularly the accumulation of certain minerals, have received considerably less attention [10]. Emerging evidence indicates that elevated concentrations of minerals such as iron, copper, or manganese may foster a pro-oxidative environment, triggering oxidative stress and neuroinflammation, as well as disruptions in cellular signaling pathways [11,12]. These processes can alter neuronal homeostasis and potentially compromise the structure and function of the nervous system, with deleterious effects on cognition.

This gap in the literature is particularly relevant in light of the globalization of dietary patterns, the widespread use of fortified foods, and increasing reliance on dietary supplements, all of which contribute to more frequent exposure to supraphysiological levels of certain minerals. In this context, the present study aimed to examine the association between dietary intake of selected minerals, with particular focus on iron and zinc, and cognitive performance in Spanish adults with obesity. Given the neurobiological relevance of these minerals and prior evidence suggesting that variations in mineral exposure may be linked to cognitive functioning, this study explored potential associations between dietary intake of iron and zinc and performance in executive-related cognitive domains, including reasoning, cognitive flexibility, and working memory.

## 2. Materials and Methods

This cross-sectional study was conducted with a sample of 230 Spanish adults, within the framework of the research project Personalized Obesity Treatment by Learning and Analysing a Multimodal Space of the Disease with an Integral Approach (Tech4Diet-Person). The overarching aim of Tech4Diet-Person is to improve obesity treatment through personalized interventions based on deep multimodal learning and continuous patient monitoring, by developing digital tools that support tailored nutrition and motivation for long-term lifestyle changes. This project represents the continuation of a research line initiated in two previous studies: 4D Modelling and Visualization of the Human Body for the Improvement of Adherence to Dietary-Nutritional Treatment of Obesity through Low-Cost Technologies (TIN2017-89069-R, Tech4Diet, 2017) and Predictive Models of the Morphological Evolution of the Human Body to Improve Adherence (PID2020-119144RB-100, Tech4Diet-Predict, 2020).

The study protocol was reviewed and approved by the Ethical Committee of the University of Alicante (File Nos. UA-2016-06-30 and UA-2021-11-18). Participants were recruited following strict ethical standards and current data protection regulations, ensuring respect for privacy, confidentiality, and the voluntary nature of participation. All individuals were fully informed about the study’s objectives, procedures, and conditions, and were reminded of their right to withdraw at any time without consequence. Written informed consent was obtained from all participants before enrolment. The signed consent forms were securely stored by the research team in accordance with institutional and ethical guidelines. No minors were included in the study. All recruitment procedures and assessments were carried out at the Faculty of Health Sciences of the University of Alicante between 10 January 2022 and 20 February 2024. Data collection included a semi-structured interview for sociodemographic information, dietary–nutritional questionnaires, and neuropsychological tests to assess current nutritional status and cognitive function. The sample size was estimated a priori using G*Power 3.1. A non-parametric comparison between two independent groups (Mann–Whitney U test) was assumed, with a two-tailed significance level (α = 0.05), statistical power (1 − β) of 0.90, and an anticipated small effect size (d = 0.25), consistent with expected effect magnitudes in nutritional epidemiology research. Under these assumptions, the required minimum sample size was estimated at 130 participants.

Inclusion criteria were being an adult between 18 and 65 years of age and having Spanish as a native language. Exclusion criteria included: (i) the presence of a professionally monitored endocrine–metabolic disorder; (ii) a history of neurological disease or acquired brain injury (e.g., stroke, traumatic brain injury, or neurodegenerative conditions such as Parkinson’s disease); (iii) a history of severe psychopathology according to DSM-5 criteria; (iv) current psychiatric treatment; and (v) pregnancy and/or lactation in women.

### 2.1. Sociodemographic and Lifestyle Variables

Sociodemographic variables were collected through a specific semi-structured interview, which included questions regarding the following aspects: sex (female, male), age (years), body mass index (BMI), marital status (single, married/in a relationship), educational level (primary, secondary, university), and employment status (employed, unemployed).

### 2.2. Cognitive Assessment

The General Cognitive Assessment Battery (CAB) from CogniFit was used, a scientifically validated computerized neuropsychological tool that evaluates multiple cognitive domains through standardized tasks [13]. The CogniFit battery has been widely applied in both clinical and research contexts, as its component tests have been validated against various standardized neuropsychological assessments [14]. Moreover, numerous studies have employed CogniFit’s cognitive activities with healthy populations (including children, adults, and older adults) with the aim of assessing or enhancing cognitive functioning. These studies are characterized by high methodological rigor, granting CogniFit a robust level of empirical evidence in the field of cognitive interventions [15,16].

The CAB is administered individually via a digital device (computer, mobile phone, or tablet) and takes approximately 40 min to complete. The system generates a comprehensive report consisting of three sections: well-being indicators (physical, psychological, and social well-being), cognitive profile, and conclusions (including a description of different indices, specific recommendations, and a personalized action plan).

**Figure 1:**
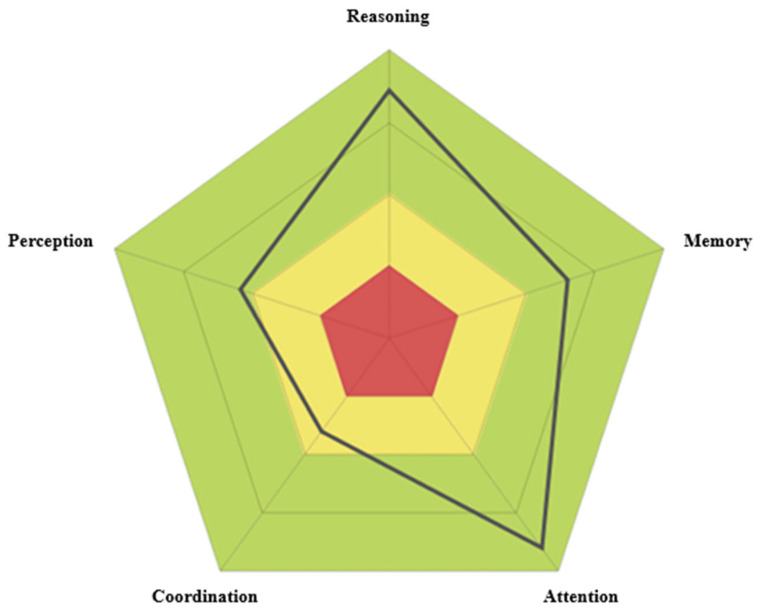
Cognitive profile circular diagram (CAB—CogniFit).

The cognitive profile is presented in a circular diagram for each evaluated domain (Figure 1), providing normalized quantitative scores based on a large population database, adjusted for the participant’s age and sex [14]. Although CogniFit scores are calculated in percentiles, they are presented on a unified scale ranging from 0 to 800. In this scale, higher scores reflect better cognitive performance. Scores between 0 and 200 (red zone) indicate performance significantly below the expected average, interpreted as a cognitive weakness in the assessed domain. Scores between 200 and 400 (yellow zone) reflect performance below the average, suggesting optimizable cognitive abilities. Scores between 400 and 600 (first green zone) correspond to adequate cognitive performance, indicating that the assessed ability is within the normative range. Finally, scores above 600 (second green zone) are considered cognitive strengths, as they reflect above-average functioning.

The total cognitive profile score was calculated as the mean of five core cognitive domains: reasoning, memory, attention, coordination, and perception. Each of these domains is composed of the average performance across several basic cognitive functions. For the purposes of this study, only selected functions were analyzed, as defined in the report generated by the CogniFit platform:

- Reasoning: Defined as the ability to efficiently process acquired information. It evaluates executive function and is composed of the following sub-functions:
- Planning: The ability to mentally anticipate the correct way to execute a task.
- Processing speed: The amount of time it takes an individual to complete a mental task.
- Cognitive flexibility: The brain’s capacity to adapt behavior and thinking to novel, changing, or unexpected situations.
- Working memory: This function refers to the ability to temporarily store and manipulate the necessary information for performing complex cognitive tasks. Although the CogniFit platform categorizes it under the domain of memory, the scientific literature widely recognizes it as an executive function due to its anatomical localization in the prefrontal cortex [17] and its involvement in cognitive control and decision-making processes [18]. For this reason, and to encompass a comprehensive analysis of executive functions, working memory was included in this study as part of the executive function category.

### 2.3. Dietary Intake Assessment

To evaluate dietary intake, a semi-quantitative Food Frequency Questionnaire (FFQ) comprising 93 items was used. This instrument was specifically developed for the Nutrition and Health Survey of the Valencian Community (ENCV) and has been validated and employed in several epidemiological studies involving Spanish adult populations [19].

The questionnaire gathered detailed information on the frequency and usual quantity of consumption of various food groups, including bread, fats, meats, dairy products, and beverages. Consumption frequency was recorded based on the participants’ intake over the year preceding the assessment. Response options ranged from “once a day” to “more than six times a day” for daily intake; “once a week”, “2–4 times per week”, or “5–6 times per week” for weekly intake; and “1–3 times per month” or “occasional/none” for monthly intake. For seasonal foods, such as fruits and vegetables, the questionnaire specified the frequency during their natural season.

Standardized portion sizes were predefined within the FFQ (e.g., one apple corresponding to approximately 150 g), and participants reported consumption frequency based on these reference units rather than providing self-estimated quantities. Mineral intake was subsequently calculated by linking these standardized portion sizes to the nutrient composition data provided by the Spanish Food Composition Database (BEDCA), which includes the full range of food items assessed in the questionnaire.

The estimation of mineral intake was therefore based exclusively on dietary sources derived from food consumption as assessed by the FFQ. Information on the use of dietary supplements or fortified products was not systematically collected and was consequently not included in the nutrient intake calculations.

### 2.4. Statistical Analysis

Frequencies and percentages were calculated for categorical variables, both overall and stratified by groups. Categorical variables were compared across groups using the Chi-square test (χ^2^), and effect sizes were assessed using Cramér’s V (with >0.1 considered small, >0.3 moderate, and >0.5 large) [20]. Quantitative variables were described using means and standard deviations (SDs), both globally and disaggregated by groups. Group differences were analyzed using the non-parametric Mann–Whitney U test, and effect sizes were assessed using Rosenthal’s r (with >0.1 considered small, >0.3 moderate, and >0.5 large) [21].

To quantify dietary intake, the frequencies reported in the FFQ were converted into daily frequencies. This conversion involved assigning an average numerical value to each response category: for example, “never or rarely” was translated to 0.0; “1–3 times per month” to 0.07 (2/30); “once a week” to 0.14 (1/7); “2–4 times per week” to 0.42 (3/7); “5–6 times per week” to 0.78 (5.5/7); and “once a day” to 1.0 (1/1). These values, representing daily consumption frequency, were multiplied by the corresponding portion size (in grams) for each food item to calculate the estimated daily intake of each food.

Once the daily intake of each food was calculated, the energy and nutrient content per portion was estimated using the Spanish Food Composition Database (Red BEDCA) [22], considering only the edible portion. Subsequently, total individual intake of energy and nutrients was obtained by summing the values for all food items.

To compare dietary intake between groups, standardized z-scores were calculated for each cognitive function and interpreted according to standard normal distribution criteria. A threshold of −1.5 standard deviations was used to define low cognitive performance, in line with commonly adopted neuropsychological conventions for identifying clinically meaningful deviations from normative functioning in population-based research [23]. Based on this criterion, two groups were established for each cognitive function analyzed: total Cognifit score, reasoning, cognitive flexibility, processing speed, planning, and working memory. Following group segmentation, the Mann–Whitney U test was applied to compare differences in mineral intake between groups. Given the number of comparisons performed across minerals and cognitive domains, false discovery rate correction was applied using the Benjamini–Hochberg procedure to control for the risk of type I error. Statistical significance was initially set at α = 0.05, and effect size was calculated using the r statistic, where 0.10 indicates a small effect, 0.30 medium, and 0.50 large [20].

To further examine the association between mineral intake and cognitive performance, separate binary logistic regression analyses were conducted for cognitive domains that showed nominal differences in mineral intake between groups. For each model, low performance in the corresponding cognitive domain (reasoning, cognitive flexibility, or working memory) was used as the dependent variable (coded as 0 = normal, 1 = low). Independent variables included the relevant mineral intake (iron for reasoning and cognitive flexibility, and zinc for working memory), along with age, body mass index (BMI), total daily energy intake, and educational level as covariates. All predictors were entered simultaneously using the enter method to estimate their independent contribution to the likelihood of low cognitive performance. Regression coefficients (B), standard errors, Wald statistics, *p*-values, odds ratios (Exp(B)), and 95% confidence intervals were reported.

All statistical analyses were performed using IBM SPSS Statistics for Windows, version 21.0 (IBM Corp., Armonk, NY, USA; 2012). Values of *p* < 0.05 were considered statistically significant.

## 3. Results

The sample consisted of 230 Spanish adults, with a mean age of 45.91 years (SD = 9.92), including 86 men (37.4%) and 144 women (62.6%). Most participants were married or in a stable partnership (64.8%), had completed higher education (52.2%), and were currently employed (84.3%).

As shown in Table 1, no significant sex differences were observed in age (*p* = 0.400; r = 0.06), body mass index (*p* = 0.452; r = 0.05), or total daily energy intake (*p* = 0.129; r = 0.10). However, body weight differed significantly between men and women, with men presenting higher mean weight values than women (*p* = 0.001; r = 0.27), reflecting a small-to-moderate effect size.

Regarding sociodemographic characteristics, no statistically significant differences were found between men and women in marital status (*p* = 0.221; V = 0.081) or educational level (*p* = 0.202; V = 0.118). In contrast, significant sex differences were observed in employment status (*p* = 0.001; V = 0.234), with a higher proportion of unemployment among women and a higher proportion of employment among men, corresponding to a small-to-moderate effect size.

Table 2 presents differences in daily mineral intake according to cognitive performance across the assessed domains. These between-group comparisons are based on unadjusted Mann–Whitney tests and should therefore be interpreted as descriptive, as they do not account for potential confounding factors such as BMI. Higher dietary iron intake was observed in participants with low performance compared to those with normal performance in specific executive-related domains. In particular, iron intake was higher in the low-performance group for reasoning (23.38 ± 5.79 mg vs. 20.68 ± 6.23 mg; *p* = 0.039; r = 0.14) and cognitive flexibility (22.98 ± 6.28 mg vs. 20.61 ± 6.19 mg; *p* = 0.038; r = 0.14). A similar pattern was observed for working memory (24.14 ± 8.68 mg vs. 20.55 ± 5.87 mg; *p* = 0.074; r = 0.12). No differences were observed in iron intake for total score, processing speed, or planning.

Additionally, higher zinc intake was observed in the low working memory performance group compared with the normal performance group (14.75 ± 5.00 mg vs. 12.53 ± 3.42 mg; *p* = 0.025; r = 0.15). For the remaining cognitive domains, no differences in zinc intake were observed. Across all comparisons, effect sizes were small (r < 0.20), indicating modest differences between groups.

However, after applying false discovery rate correction using the Benjamini–Hochberg procedure to account for multiple comparisons across minerals and cognitive domains, none of the observed associations remained statistically significant. Accordingly, these findings should be interpreted as exploratory and hypothesis-generating.

Table 3 presents the results of the binary logistic regression analyses examining the association between mineral intake and low performance across specific executive cognitive domains. In the model predicting low reasoning performance, higher iron intake remained significantly associated with an increased likelihood of low performance after adjustment for age, educational level, and total daily energy intake (B = 0.223; *p* = 0.006; OR = 1.25; 95% CI: 1.07–1.47). Total energy intake was also significantly associated with reasoning performance (B = −0.002; *p* = 0.025). Educational level was a significant predictor in the overall model (*p* = 0.008), with both secondary and higher education associated with lower odds of low reasoning performance compared with primary education.

In contrast, in the model for cognitive flexibility, iron intake was not significantly associated with low performance after adjustment (*p* = 0.131). However, age remained a significant predictor (B = 0.057; *p* = 0.038), and educational level was again significantly associated with cognitive flexibility (*p* = 0.034), indicating lower odds of low performance among participants with higher educational attainment.

For working memory, higher zinc intake was independently associated with an increased likelihood of low performance after adjustment (B = 0.309; *p* = 0.024; OR = 1.36; 95% CI: 1.05–1.79). Educational level showed a strong overall association with working memory performance (*p* < 0.001), with both secondary and higher education levels linked to substantially lower odds of low performance compared with primary education. Neither age nor total energy intake were significant predictors in this model.

## 4. Discussion

The aim of this study was to examine the association between dietary intake of selected minerals, with particular focus on iron and zinc, and cognitive performance in Spanish adults with obesity. The findings suggested patterns in which higher self-reported intake tended to be observed among individuals with lower performance in specific executive-related cognitive domains, particularly reasoning, cognitive flexibility, and working memory, even after adjustment for age, body mass index (BMI), total daily energy intake, and educational level. These results contribute to the growing body of literature suggesting that not only mineral deficiencies but also higher levels of intake may be linked to variations in cognitive functioning.

Evidence from the present study suggests that individuals with lower cognitive performance reported, on average, higher iron intake, with differences observed specifically in the domains of reasoning and cognitive flexibility. These findings may indicate a potential relationship between higher self-reported dietary iron intake and certain aspects of cognitive performance. However, the cross-sectional nature of the study and the reliance on dietary self-report preclude causal interpretation. Importantly, the existing mechanistic literature on iron and brain function largely addresses tissue-level or systemic iron dysregulation rather than dietary intake as assessed through food frequency questionnaires. The relationship between reported dietary intake and iron availability in the central nervous system is complex and regulated by multiple physiological processes, including intestinal absorption, systemic transport, storage mechanisms, and metabolic regulation [24,25]. Consequently, the present results should not be interpreted as reflecting alterations in brain iron accumulation or as indicative of neurodegenerative mechanisms.

Previous research has described associations between altered iron homeostasis and cognitive decline [26], and several neurobiological pathways have been proposed to contextualize these observations. One of the most frequently discussed mechanisms involves oxidative stress, as iron can participate in redox reactions that generate reactive oxygen species, potentially contributing to cellular damage affecting lipids, proteins, and DNA. Such processes have been discussed in relation to brain regions involved in higher-order cognitive functions, including the hippocampus and prefrontal cortex [27]. Other proposed mechanisms include neuroinflammatory responses, as iron may modulate microglial activation and the expression of pro-inflammatory cytokines such as IL-6 and TNF-α [28]. Alterations in synaptic plasticity have also been suggested, possibly related to disruptions in calcium homeostasis and neurotransmitter signaling pathways, including glutamatergic and dopaminergic systems [29]. In addition, structural brain differences, including alterations in grey matter and changes in functional connectivity, have been described in relation to variations in iron regulation [30]. Such observations have also been reported in the context of neurodegenerative conditions, including Alzheimer’s and Parkinson’s disease, where altered iron homeostasis has been associated with increased regional iron concentrations, oxidative modifications of tau protein, pathways related to its phosphorylation, and interactions with alpha-synuclein [31,32,33]. These mechanistic frameworks may provide biological context for understanding potential links between iron regulation and brain function. However, they derive primarily from experimental or clinical models. Therefore, their relevance should be interpreted cautiously in studies based on self-reported dietary intake, such as the present investigation.

A statistically significant association was observed between higher self-reported zinc intake and working memory performance, although the effect size was small. This pattern may be consistent with previous literature describing zinc as a neuroactive micronutrient involved in multiple aspects of synaptic function. Experimental studies have suggested that zinc participates in glutamatergic neurotransmission by modulating NMDA and AMPA receptor activity and contributing to synaptic plasticity in regions such as the hippocampus and prefrontal cortex [34]. At the same time, alterations in zinc homeostasis, particularly at elevated extracellular concentrations, have been associated with disruptions in calcium regulation, mitochondrial dysfunction, and increased oxidative stress, processes that may affect neuronal viability [35]. Such mechanisms have been discussed as potential contributors to changes in synaptic dynamics relevant to cognitive functions including attention and working memory [36]. However, it is important to distinguish between mechanistic evidence derived from experimental or clinical models and the assessment of dietary intake in population-based studies. Therefore, the present findings should be interpreted cautiously and not as indicative of altered brain zinc accumulation or direct neurotoxic effects.

Cognitive functioning, particularly executive processes, may be shaped by a range of sociodemographic and modifiable lifestyle factors, including mechanisms related to information processing and perception–action dynamics [37]. Accordingly, multivariable logistic regression analyses were performed to further explore the domain-specific patterns identified in the descriptive comparisons. After adjustment for age, body mass index, total energy intake, and educational level, higher self-reported iron intake remained significantly associated with increased odds of low reasoning performance, suggesting that this relationship persisted after accounting for key sociodemographic and dietary covariates. In contrast, the relationship between iron intake and cognitive flexibility was no longer statistically significant in the adjusted model, indicating that the unadjusted association may have been influenced by residual confounding or limited statistical power, which are common challenges in observational nutritional research [38]. For working memory, higher self-reported zinc intake remained significantly associated with greater odds of low performance after adjustment for relevant sociodemographic and nutritional variables. Despite the modest effect size, this pattern indicates that the association persisted after multivariable adjustment, although it should be interpreted cautiously given the observational design. Across models, educational attainment consistently emerged as a strong protective factor, supporting the well-established role of cognitive reserve and socioeconomic determinants in shaping executive functioning outcomes and resilience to cognitive decline [39,40]. Age showed a weaker and domain-specific association, while body mass index and total energy intake did not demonstrate consistent relationships with cognitive performance. Overall, these findings reinforce the exploratory nature of the observed associations and highlight the importance of multivariable modeling when examining potential links between dietary factors and cognitive functioning in population-based studies. Although the clinical implications remain uncertain due to the observational design and modest effect sizes, the results may contribute to a more nuanced understanding of how patterns of dietary mineral intake could be considered within broader nutritional and preventive approaches to cognitive health in individuals with obesity. In this context, attention may be warranted when evaluating dietary patterns characterized by a relatively high consumption of iron- and zinc-rich foods, such as red meat, seafood, legumes, dairy products, and leafy green vegetables, particularly among individuals with lower cognitive performance.

Despite the relevance of these findings, several methodological and analytical limitations should be considered when interpreting the results. First, the cross-sectional design precludes causal inference regarding the relationship between mineral intake and cognitive performance. As such, the observed associations should be interpreted as exploratory rather than indicative of directional or mechanistic effects. Second, dietary intake was assessed using a self-reported food frequency questionnaire (FFQ), which may be subject to recall bias and reporting inaccuracies. Moreover, information on the use of dietary supplements or fortified foods was not available, meaning that estimated intake may not fully reflect total mineral exposure, particularly when considering patterns of higher intake. Third, the absence of biochemical measures, such as serum ferritin or circulating zinc concentrations, limits the ability to determine systemic mineral status or to confirm whether reported intake corresponds to physiological imbalance. Finally, although the overall sample size was adequate for the main analyses, the subsamples with lower cognitive performance were comparatively small, which may affect the precision of estimates. In addition, the findings should be interpreted as primarily applicable to adults with obesity and elevated metabolic risk, rather than to the general adult population.

Future research should address these limitations by adopting longitudinal designs, incorporating objective biomarkers of mineral status to complement self-reported intake, and including larger samples of individuals with lower cognitive performance to improve the robustness and domain-specific interpretation of the observed associations.

## 5. Conclusions

Overall, the present findings suggest that higher self-reported intake of iron and zinc was nominally associated with lower performance in specific executive-related cognitive domains, particularly reasoning and working memory. These associations should be interpreted cautiously given the cross-sectional design, reliance on dietary self-report, and absence of biomarker data. The results do not allow conclusions regarding causal or neurobiological mechanisms but may contribute to a more nuanced understanding of the potential role of dietary mineral exposure in cognitive functioning among adults with obesity. Further longitudinal studies integrating objective measures of micronutrient status are required to clarify the nature and clinical relevance of these relationships.

## Figures and Tables

**Table 1:** Sociodemographic characteristics of the sample (N = 230).

Variables	Total (N = 230)	Male (N = 86)	Female (N = 144)	*p*	ES
Age, mean (SD)	45.91 (9.92)	45.17 (9.73)	46.35 (10.04)	0.400	0.056
BMI	31.804 (6.200)	31.018 (6.580)	32.100 (6.485)	0.452	0.050
Weight	88.406 (19.251)	95.674 (19.390)	84.065 (17.868)	**0.001**	**0.271**
Total energy intake	2906.31 (849.65)	3004.07 (897.78)	2847.92 (817.19)	0.129	0.100
Marital Status, % (*n*)					
Single	35.2 (81)	30.2 (26)	38.2 (55)	0.221	0.081
Married/In a relationship	64.8 (149)	69.8 (60)	61.8 (89)		
Education level, % (*n*)					
Primary	9.6 (22)	14.0 (12)	6.9 (10)	0.202	0.118
Secondary	38.3 (88)	34.9 (30)	40.3 (58)		
University	52.2 (120)	51.2 (44)	52.8 (76)		
Employment status, % (*n*)					
Unemployed	15.7 (36)	4.7 (4)	22.2 (32)	**0.001**	**0.234**
Employed	84.3 (194)	95.3 (82)	77.8 (112)		

Note. *p* values correspond to chi-square tests for categorical variables and Mann–Whitney U tests for continuous variables. ES = Effect size; SD: standard deviation; BMI: body mass index; for continuous variables, Rosenthal’s r is reported; for categorical variables, Cramér’s V is used. Bolded *p* values indicate statistical significance (*p* < 0.05).

**Table 2:** (A) Daily mineral intake (mg/day) by cognitive performance in total score, reasoning, and cognitive flexibility. (B) Daily mineral intake (mg/day) by cognitive performance in processing speed, planning, and working memory.

**(A)**									
**Mineral**	**Total Cognifit Score**			**Reasoning**			**Cognitive Flexibility**		
	**Normal (*n* = 213)**	**Low (*n* = 17)**	***p*** **/r**	**Normal (*n* = 215)**	**Low (*n* = 15)**	***p*** **/r**	**Normal (*n* = 206)**	**Low (*n* = 24)**	***p*** **/r**
Calcium	1146.26 (398.65)	1162.84 (710.52)	0.290/0.07	1153.94 (432.72)	1055.04 (334.30)	0.428/0.05	1143.35 (431.63)	1183.01 (392.63)	0.584/0.04
Iron	20.69 (6.10)	23.01 (7.50)	0.196/0.09	**20.68 (6.23)**	**23.38 (5.79)**	**0.039/0.14**	**20.61 (6.19)**	**22.98 (6.28)**	**0.038/0.14**
Potassium	3966.15 (1106.08)	4085.55 (1220.97)	0.866/0.01	3963.50 (1126.24)	4139.41 (909.01)	0.330/0.06	3946.54 (1100.65)	4219.02 (1206.88)	0.203/0.08
Magnesium	429.44 (114.70)	455.48 (142.98)	0.368/0.06	429.31 (118.22)	460.86 (93.27)	0.091/0.11	428.35 (114.86)	457.28 (132.56)	0.166/0.09
Sodium	9237.89 (4294.38)	9542.84 (3942.12)	0.810/0.02	9283.60 (4303.50)	8928.27 (3729.06)	0.884/0.01	9279.19 (4284.42)	9099.42 (4149.49)	0.826/0.02
Phosphorus	1925.61 (534.58)	1997.32 (749.46)	0.971/0.00	1931.08 (557.53)	1928.43 (472.96)	0.738/0.02	1919.66 (550.29)	2027.46 (564.31)	0.220/0.08
Iodine	268.59 (86.34)	255.34 (77.21)	0.466/0.05	269.25 (86.60)	244.11 (68.00)	0.245/0.08	268.98 (84.24)	255.88 (97.85)	0.454/0.05
Selenium	127.26 (46.15)	130.58 (59.88)	0.992/0.00	127.66 (47.70)	125.39 (39.63)	0.744/0.02	125.89 (46.40)	141.43 (52.13)	0.102/0.11
Zinc	12.63 (3.50)	13.85 (4.97)	0.303/0.07	12.68 (3.66)	13.27 (3.09)	0.224/0.08	12.61 (3.61)	13.65 (3.73)	0.084/0.11
**(B)**									
**Mineral**	**Processing Speed**			**Planning**			**Working Memory**		
	**Normal (*n* = 217)**	**Low (*n* = 13)**	***p*** **/r**	**Normal (*n* = 204)**	**Low (*n* = 26)**	***p*** **/r**	**Normal (*n* = 210)**	**Low (*n* = 20)**	***p*** **/r**
Calcium	1142.01 (398.54)	1239.00 (782.53)	0.685/0.03	1158.88 (436.23)	1058.09 (341.04)	0.237/0.08	1140.79 (401.24)	1217.78 (649.56)	0.888/0.01
Iron	20.76 (6.06)	22.55 (8.63)	0.363/0.06	20.79 (6.12)	21.40 (7.10)	0.664/0.03	20.55 (5.87)	24.14 (8.68)	0.074/0.12
Potassium	3965.25 (1091.19)	4137.25 (1468.80)	0.795/0.02	3980.74 (1125.01)	3929.72 (1028.70)	0.822/0.02	3947.80 (1092.27)	4260.34 (1304.03)	0.435/0.05
Magnesium	428.69 (113.60)	476.06 (161.15)	0.214/0.08	431.37 (117.66)	431.36 (112.46)	0.831/0.01	428.71 (114.23)	459.31 (141.89)	0.351/0.06
Sodium	9203.59 (4306.09)	10,209.25 (3442.14)	0.380/0.06	9177.55 (4286.34)	9910.70 (4087.52)	0.396/0.06	9264.91 (4294.03)	9213.36 (4013.20)	0.869/0.01
Phosphorus	1926.46 (534.81)	2005.22 (806.02)	0.937/0.01	1937.46 (540.48)	1879.52 (641.08)	0.345/0.06	1910.97 (518.52)	2140.30 (811.10)	0.288/0.07
Iodine	267.23 (86.39)	274.06 (74.12)	0.752/0.02	266.29 (87.34)	277.99 (71.19)	0.297/0.07	268.10 (86.34)	262.49 (79.48)	0.722/0.02
Selenium	127.52 (46.96)	127.38 (52.17)	0.974/0.00	128.12 (44.75)	122.73 (63.80)	0.188/0.09	125.98 (43.35)	143.60 (76.30)	0.620/0.03
Zinc	12.65 (3.50)	13.96 (5.32)	0.390/0.06	12.74 (3.61)	12.60 (3.80)	0.862/0.01	**12.53 (3.42)**	**14.75 (5.00)**	**0.025/0.15**

Note: Values are expressed as mean (SD) in mg/day. *p* values were obtained using the Mann–Whitney U test. r refers to Rosenthal’s effect size (small ≥ 0.10, moderate ≥ 0.30, large ≥ 0.50). Bolded *p* values indicate statistical significance (*p* < 0.05). “Low” performance was defined as a z-score < −1.5 in each specific cognitive domain.

**Table 3:** Binary logistic regression models for low executive cognitive performance.

**Model 1. Low Reasoning Performance**						
**Variable**	**B**	**SE**	**Wald**	***p***	**OR**	**95% CI**
Iron intake	0.223	0.081	7.654	**0.006**	1.25	1.07–1.47
Age	0.072	0.038	3.540	0.060	1.08	1.00–1.16
BMI	0.009	0.044	0.040	0.841	1.01	0.93–1.10
Total energy intake	−0.002	0.001	5.049	**0.024**	1.00	0.996–1.000
Education level (Primary)			9.703	**0.008**		
Secondary	−1.682	0.718	5.565	**0.018**	0.19	0.05–0.75
Higher	−2.339	0.807	8.756	**0.003**	0.09	0.02–0.45
**Model 2. Low Cognitive Flexibility Performance**						
Iron intake	0.084	0.054	2.422	0.120	1.09	0.98–1.21
Age	0.055	0.027	4.101	**0.043**	1.06	1.00–1.12
BMI	0.030	0.037	0.647	**0.421**	1.03	0.96–1.11
Total energy intake	0.000	0.000	0.301	0.583	1.00	1.00–1.00
Education level (Primary)			6.739	**0.034**		
Secondary	−1.361	0.607	5.021	**0.025**	0.25	0.08–0.82
Higher	−1.281	0.622	4.243	**0.039**	0.28	0.08–0.78
**Model 3. Low Working Memory Performance**						
Zinc intake	0.309	0.137	5.115	**0.024**	1.36	1.05–1.79
Age	0.018	0.029	0.387	0.534	1.02	0.96–1.08
BMI	0.028	0.042	0.437	0.509	1.03	0.95–1.12
Total energy intake	−0.001	0.001	0.841	0.359	1.00	0.997–1.001
Education level (Primary)			14.421	**<0.001**		
Secondary	−1.400	0.589	5.657	**0.017**	0.25	0.08–0.76
Higher	−4.282	1.181	13.136	**<0.001**	0.01	0.001–0.12

Note: Separate binary logistic regression models were conducted for each cognitive domain showing significant differences in mineral intake. *p* values < 0.05 are considered statistically significant and are bolded. Reference categories: education level = primary. OR = odds ratio; CI = confidence interval.

## Data Availability

The data supporting the findings of this study are available from the corresponding author upon reasonable request. Data are not publicly available due to privacy and ethical restrictions related to the protection of participants’ personal information and compliance with institutional data protection regulations.

## Abbreviations

The following abbreviations are used in this manuscript:

BMI	Body Mass Index
CAB	Cognitive Assessment Battery
CRP	C-reactive Protein
DSM-5	Diagnostic and Statistical Manual of Mental Disorders, Fifth Edition
ENCV	Nutrition and Health Survey of the Valencian Community
FFQ	Food Frequency Questionnaire
NHANES	National Health and Nutrition Examination Survey
OR	Odds Ratio
SD	Standard Deviation
